# Tuberculosis Dispensaries in Theory and Practice

**Published:** 1913-12-13

**Authors:** Frederick J. C. Blackmore

**Affiliations:** Senior A.M.O., Tuberculosis Centre, Birmingham


### TUBERCULOSIS DISPENSARIES IN THEORY AND PRACTICE.

By FREDERICK J. C. BLACKMORE, Senior A.M.O., Tuberculosis Centre, Birmingham.

The rapid extension of the system of tuberculosis dispensaries throughout the country has resulted—perhaps naturally—in a certain amount of criticism. This is to be welcomed, as it affords an opportunity of emphasising the need of these special dispensaries, and also of demonstrating how they are able to deal with tuberculosis in a way which is not possible for other agencies. Indeed it has been stated that the work can be done equally well and efficiently by the out-patient departments of our hospitals. This statement indicates misapprehension, and has probably been prompted by a fear that the establishment of dispensaries will seriously deplete the attendance in the out-patient departments, and that the training of medical students in tuberculosis will pass out of the hands of our teaching hospitals and become part of the duties of the dispensary staff in the same way that the practical teaching of infectious diseases devolves upon the medical staff of our isolation hospitals.

It will be well, therefore, to consider the principles underlying the establishment of dispensaries, their method of working, and to see how in practice they differ from out-patient departments. Tuberculosis is an infective and endemic disease responsible for an appalling mortality. It cannot be successfully treated without an organisation so constituted that it is able to grapple with every aspect of the subject and follow up its various ramifications throughout the social cosmos. This is what the tuberculosis dispensary is able to do and what the ordinary out-patient department of our hospitals cannot do. To be entirely successful the dispensary must of necessity have the closest co-operation of all other institutions. It works by—

1. Acting as the headquarters of the organisation—disseminating information and advice as to the best means of treatment and prevention. Professor Calmette prefers to call the dispensary a preventorium as more descriptive of its chief function.
2. Examining all notified cases of the disease and acting as a "clearing-house" for such patients.
3. Treating patients after their discharge from sanatoria and other institutions, and in certain cases undertaking all the medical treatment of those who do not need institutional treatment or who are unable to absent themselves from work for long.
4. Carrying out an energetic search for unsuspected and early cases. This means a thorough examination of all "contacts."
5. Providing, in co-operation with the general practitioner, home treatment for the mass of tuberculous patients. Tuberculin is provided if necessary, and periodical visits are paid to discuss with the patients' own doctors the lines of treatment.
6. Co-operation with the Public Health Service. In most places the Tuberculosis Service will be under the direct administrative control of the medical officer of health, with beneficial results as regards housing and sanitary deficiencies met with in the course of the home visiting.
7. Home visiting by trained nurses to secure hygienic and dietetic measures.
8. Co-operation with all the philanthropic and charitable agencies of the district. In many places it will be advantageous to establish special provident and after-care committees.

It is obvious that as at present constituted the out-patient departments of the hospitals are not able to undertake the duties indicated under the headings 4 to 8. Critics of the dispensaries suggest that they do not carry out the work thoroughly in the ways suggested. It is, however, not to be expected, in the short time that has elapsed since most of the dispensaries in England were established, that a complete and detailed scheme fulfilling the above requirements could be organised. Those dispensaries which have been and still are maintained by voluntary associations have an advantage in that their expenditure is entirely controlled by their own committees who fully realise the needs of their institutions, and are anxious that they reach their ideals as early as possible. This is not entirely the case where municipalities control and finance the dispensaries. All Public Health committees have not yet learned to regard tuberculosis with that broad outlook which is essential if successful results are to be obtained. Furthermore, municipal authorities are always faced by the ogre of rising rates. Municipal dispensaries cannot attain their full usefulness until it is realised that money spent on this work is money well spent, in that it will eventually lessen the enormous amount now spent on Poor-Law relief and the loss to the community caused by the sickness and death of so many wage-earners in the prime of life. This question of funds applies equally well to the hospitals which are now constantly hampered in their work by debt. Gradual evolution is the only road by which tuberculosis dispensaries can attain their ideals; the loyal co-operation of existing institutions and voluntary bodies will be of material assistance in the struggle against adverse circumstances. It is probable that eventually more drastic powers will have to be conferred on our Public Health authorities, *e.g.*, the power to remove such cases as are a menace to the health of the community. Tramps, homeless persons in an advanced stage of the disease, patients living in overcrowded quarters, those who are unable or unwilling to follow sanitary instructions—and the last class is no small one—cannot be allowed to undo at one end what the dispensary is endeavouring to do at the other end. When we consider the various agencies which are and will be used in the crusade against tuberculosis—farm colonies, open-air schools and preventoria for children, homes of rest, etc., and the various voluntary associations—it becomes increasingly evident that the hospitals cannot take the place of tuberculosis dispensaries, but can and must be valuable co-agents in a more comprehensive organisation of which the tuberculosis dispensary will be the fountain-head.

